# Healthcare utilisation and costs associated with poor access to diagnosis and treatment for children and young people with tic disorders

**DOI:** 10.1136/bmjment-2024-301241

**Published:** 2024-11-07

**Authors:** Charlotte L Hall, Marie Le Novere, Tara Murphy, Emma McNally, Christopher Hollis, Rachael Hunter

**Affiliations:** 1 NIHR MindTech MedTech Health Research Centre, Institute of Mental Health, University of Nottingham, Nottingham, UK; 2 NIHR Nottingham Biomedical Research Centre, Institute of Mental Health, University of Nottingham School of Medicine, Nottingham, UK; 3 Research Department of Primary Care and Population Health and Priment CTU, University College London, London, UK; 4 University College London Great Ormond Street Institute of Child Health, London, UK; 5 Great Ormond Street Hospital for Children NHS Foundation Trust, London, UK; 6 Tourettes Action, Farnborough, UK; 7 Child and Adolescent Psychiatry, Queen's Medical Centre, Nottingham, UK

**Keywords:** Child & adolescent psychiatry, PSYCHIATRY

## Abstract

**Background:**

There are no specific national guidelines in England to guide healthcare professionals in how to assess or treat young people with tic disorders. Access to evidence-based treatment, including behavioural therapy, is of limited availability.

**Objectives:**

This study examined the economic impact on services arising from a lack of access to appropriate healthcare services for young people with tic disorders, alongside the impact on school attendance.

**Methods:**

This study used data from the randomised controlled trial ‘ORBIT’ (Online Remote Behavioural Intervention for Tics). ORBIT compared online exposure and response prevention behavioural therapy for tics with online psychoeducation and recruited 224 young people aged 9–17 years in England. Here, we explore costs of health service use and school absenteeism from children who participated in the ORBIT trial and present these alongside the economic impact of including ORBIT within a tic service. We supplement ORBIT data with findings from two case studies.

**Findings:**

The data showed that patients have care from several healthcare professionals and miss school due to accessing care for tics. The case studies suggest that most of these contacts with specialist services are unlikely to be supportive. However, adding ORBIT could save the National Health Service £1 million.

**Conclusions:**

Young people with tic disorders are likely to engage in substantial use of healthcare resources because of inadequate care pathways. The availability of an evidence-based online therapy such as ORBIT could save money to the healthcare system.

**Clinical implications:**

There is a need to improve service provision and develop national guidelines for tic disorders.

**Trial registration number:**

ISRCTN70758207, NCT03483493.

**WHAT IS ALREADY KNOWN ON THIS TOPIC**

- Most young people in England with tic disorder cannot access evidence-based behavioural therapy and there are no national guidelines in how to assess and treat tics.

**WHAT THIS STUDY ADDS**

- The lack of adequate service provision for tic disorders results in unnecessary cost to the National Health Service and time off school for young people. Adding an evidence-based online therapy service could save money and benefit patients.

**HOW THIS STUDY MIGHT AFFECT RESEARCH, PRACTICE OR POLICY**

- Providing guidelines to support healthcare professionals in assessing and treating tic disorders may standardise practice and improve tic care pathways. Providing access to evidence-based therapy is required to reduce burden on existing healthcare services that are not resourced to treat tics.

## Background

 Tic disorders, including Tourette syndrome (TS) and chronic tic disorders (CTDs), are defined by involuntary and repetitive motor and vocal tics that have been present for at least a year.^1^ They are a common condition, affecting approximately 1% of young people in England, with a higher prevalence in boys than girls.^2^ Although tics may improve into adulthood, studies indicate that only one-third of individuals will experience remission within 10 years of their first tic onset.^3^ Having a tic disorder is associated with significant distress for the individual, resulting in a fourfold increase in risk of death by suicide,^4^ pain from tics^5^ and negative effects on academic, social and occupational function.^6^,^8^

There are effective treatments to support young people with tic disorders. Medication options include antipsychotics and non-adrenergic agents. However, although beneficial, the effect size of these drugs is often small, and the side effects, which include sedation and weight gain, are often intolerable.^9^ Consequently, there is growing evidence for behavioural therapy. The European clinical guidelines for TS^10 11^ and a Health Technology Assessment evidence synthesis^9^ both recommend behavioural therapy as a first-line treatment. There are three behavioural therapy approaches for tic disorders. One is habit reversal therapy (HRT), whereby patients employ a competing response (eg, an incompatible action to their tic) to prevent the tic; the second is comprehensive behavioural intervention for tics (CBIT) which combines HRT with relaxation techniques and functional analysis; and the third is exposure and response prevention (ERP), whereby patients learn to control their tics by developing a tolerance of the urge to tic.^12^ Although not a behavioural therapy, psychoeducation is also recognised as being important in supporting with tic disorders.^10 11^ However, despite research demonstrating the effectiveness of behavioural therapy for tic disorders,^13^ in the UK, it is estimated that only one in five young people can access therapy for their tics, and fewer than half receive the recommended number of sessions.^14^

In the UK, there are no National Institute for Health and Care Excellence (NICE) guidelines for the assessment, diagnosis and treatment of tic disorder. A lack of guidelines may have contributed to the variation in healthcare provision and lack of uncertainty about how best to support the patients. The impact of this was highlighted in a recent survey of patient experience of accessing healthcare for tic disorders.^15^ The findings demonstrated that respondents often felt their general practitioner (GP) was not knowledgeable on tic disorders. Respondents reported a struggle to get referred onto a secondary care service, and even when their referral was made it was declined as the service did not provide support for tic disorders, leading to multiple subsequent referrals to different services, some of which required long travel time from their home. Almost one-third of respondents reported access to private healthcare as a result of inadequate National Health Service (NHS) services.^15^

Online, remotely delivered behavioural therapy may play a key role in improving access to evidence-based care for young people with tic disorders. A recent randomised controlled trial ‘ORBIT’ (Online Remote Behavioural Intervention for Tics) recruited 224 young people with tics who were randomised to receive either 10 weeks of online, remotely delivered ERP or 10 weeks of online, remotely delivered psychoeducation. The content was delivered via the web-based chapters, and during the 10-week intervention, the young people, and their supporter (typically the parent), could access an E-coach through the online platform. The E-coach provided motivation in treatment and offered troubleshooting advice but did not provide therapeutic input. The participants aged 9–17 years were followed up to 18 months after randomisation.^16^ The findings showed that ERP was superior to psychoeducation in improving tic symptoms and this benefit was sustained up to 18 months.^16^,^18^ Health economic analysis revealed that at 18 months, the mean incremental cost per participant of the intervention compared with the control was £662 (95% CI −59 to 1384), with a mean incremental increase in quality-adjusted life-year (QALY) of 0.040 (95% CI −0.004 to 0.083) per participant. The mean incremental cost per QALY gained was £16 708.^18 19^ The results of a decision model with a 10-year time horizon show that ORBIT costs £537 less than face-to-face individual CBIT, the ‘gold standard’ treatment for tics, but results in 0.018 fewer QALYs. ORBIT had a greater than 94% chance of being cost-effective compared with individually administered CBIT for cost-effectiveness thresholds less than £20 000 per QALY gained.^19^ An embedded process evaluation of the ORBIT trial revealed that the intervention was delivered with high fidelity and was considered acceptable and valuable to young people and parents.^20^ The findings demonstrate that online therapy is a potentially clinically and cost-effective way to deliver evidence-based care to a wide range of participants, regardless of geographical location. However, ORBIT is currently not available within the NHS.

Although it is anticipated that the current poor access to evidence-based treatment and the lack of clear guidelines may have an economic impact, to date there is no published evidence exploring this. It is important that this is understood to guide future policies and make informed decisions on health infrastructure and the implementation of evidence-based interventions and care pathways.

### Objective

The aim of this study was to demonstrate the impact on services and education resulting from a lack of access to diagnosis and treatment for individuals suffering from chronic tic conditions. We do this by exploring health resource use data gained in the ORBIT trial and supplementing this with anonymous patient and public involvement case studies of their experiences in accessing healthcare for tic disorders.

## Methods

### Design

The study used findings from the ORBIT trial. The full protocol of the ORBIT trial was published.^16^ The study was prospectively registered with the ISRCTN Registry (ISRCTN70758207) and the ClinicalTrials.gov (NCT03483493). The ORBIT trial findings were supplemented by case studies of patient and public involvement members from Tourettes Action, the UK charity for tic disorders describing their healthcare journeys.

### Participants

A total of 224 participants took part in the ORBIT trial. Participants from both arms of the trial were included in this study. The published protocol contains the further details,^16^ and in subsequent papers,^17 18^ however, eligible participants were aged 9–17 years with a moderate/severe tic disorder (TS or CTD) defined as score of >15 on the Yale Global Tic Severity Scale–Total Tic Severity Score (YGTSS-TTSS),^21^ or >10 if only motor or vocal tics were present. The main exclusion criteria were engaging in a behavioural intervention for tics in the past 12 months, starting or stopping tic medication within the previous 2 months and having suspected moderate/severe intellectual disability. If participants were under 16 years, parent/guardian consent was required along with the young person’s assent. If the participant was 16 years or older, then both parent/guardian and young person provided consent. We also provide two anonymous case studies who did not participate in the ORBIT trial.

### Measures

#### Child and Adolescent Service Use Schedule

The ORBIT trial collected information on participant healthcare resource use at baseline, and 3, 6, 12 and 18 months during the trial.^18^ This was collected using a modified version of the Child and Adolescent Service Use Schedule (CA-SUS)^22^ developed in previous studies. The CA-SUS included items related to use of specialist services and school attendance.

#### Yale Global Tic Severity Scale

The primary outcome measure used in the ORBIT intervention was the YGTSS-TTSS.^21^ The YGTSS scores the severity of motor and vocal tics by evaluating the number, frequency, intensity, complexity and interference of tics. The total motor and total vocal tic scores range from 0 to 25, which when combined give the TTSS range of 0–50. Higher scores indicate greater severity.

### Case study data

For the case studies, participants provided a timeline of service use in a table (see the online supplemental appendix), which was cross-referenced against letters from the healthcare provider where possible.

### Analysis

We present data combined from the 3 and 6-month timepoints which collected information for the previous 3 months to create consistent 6-month intervals with the 12 and 18 months of follow-up points.

#### The cost of specialist tic services

Specialist tic services include any appointments which arise as a result of the individual’s tic condition including attendance at a specialist tic clinic, Child and Adolescent Mental Health Services (CAMHS), neurology and more, as detailed in table 1. We report the number and proportion of individuals who accessed a type of service alongside the mean (SD) number of appointments for those who used the service over the course of ORBIT trial. The cost of specialist tic services from a health and social care cost perspective was calculated using the 2020 unit costs of Health and Social Care from the Personal Social Services Research Unit (PSSRU) and NHS reference costs. We report the mean total cost of specialist services at baseline, and 6, 12 and 18 months during the course of the ORBIT trial. The relationship between cost and tic severity was estimated using linear mixed effects modelling, treating the cost data at baseline to 18 months as panel data, to calculate adjusted mean costs for each level of tic severity. Means were adjusted for age, comorbidities (attention deficit hyperactivity disorder (ADHD) and obsessive-compulsive disorder (OCD)) and gender, with the individual included as a random effect.

**Table 1:** Use of specialist services related to tics at baseline

Specialist tic service	Number using service (%)	Mean number of contacts for those who have used the service (SD)
Specialist tic clinic	14/224 (6)	1 (0)
CAMHS	62/224 (28)	2 (3)
Hospital paediatrician	33/224 (15)	1 (1)
Community paediatrician	10/224 (4)	1 (0)
Neurologist	6/224 (3)	1 (0)
Psychologist	3/224 (1)	4 (5)
Speech and language therapist	5/224 (2)	1 (0)
Occupational therapists	4/224 (2)	2 (2)
Total contacts	112/224 (50)	2 (2)

CAMHS, Child and Adolescent Mental Health Services.

#### School absenteeism

Individuals in the ORBIT trial reported the number of days they were absent from school in total, as well as how many of these days off they attributed to their tic condition. We report the number and proportion of those in the trial who took days off over 6 months. For those who had at least one absent day, we report the mean (SD) number of days off. Linear mixed effects modelling adjusting for age, comorbidities and gender was used to estimate the adjusted mean number of days absent from school by tic severity.

#### Case studies of patient experiences

Two families agreed to provide an anonymous account of their experience in trying to reach a diagnosis and treatment for their child with a chronic tic condition. Their accounts are found in the online supplemental appendix and have been left in their own words apart from where names or pronouns have been replaced. For each appointment recounted in the case study, an estimated cost has been provided from the PSSRU or NHS reference costs. We report an estimated total cost for each of the case studies as well as a breakdown of what a potential streamlined pathway for tic diagnosis and treatment could look like.

## Findings

A full description of participant characteristics recruited to the ORBIT trial has been previously published.^16^,^18^ Participants had a mean age of 12 years, were predominately male (177/224; 79%) and defined their ethnicity as white (195/224; 87%). The most commonly reported comorbid disorders were anxiety (61/224; 27%), ADHD (51/224; 23%) and oppositional defiant disorder (49/221; 22%).

### Cost of specialist tic services

Table 1 shows the number of participants who used specialist services related to tics at baseline and the mean number of contacts of those who used each type of service. The table shows that 50% of participants used at least one specialist service and had an average of two appointments.

The costs of specialist tic services over the course of the ORBIT trial are shown in table 2, split into 6-month time periods. These data were used to estimate a mean additional cost per 1-point increase in the YGTSS-TTSS. Using a mixed effects regression adjusting for age, comorbidities (ADHD and OCD) and gender, the model predicted an additional £4.09 (95% CI 1.70 to 6.49) cost to the NHS on specialist services related to tics for each 1-point increase in the YGTSS-TTSS.

**Table 2:** Costs of specialist services over the course of the trial

n	Mean cost of specialist services in £ (SD)
Baseline	224	110 (212)
6 months	204	337 (590)
12 months	177	180 (408)
18 months	176	124 (255)
Total	158	614 (1001)

The ORBIT trial found an initial reduction of 4.5 points from providing online, remotely delivered ERP; this was maintained to 2.01 after 18 months during the long-term follow-up.^18^ Thus, for each 2-point reduction in the YGTSS-TTSS, we would anticipate a saving per person of £8.18 (£4.09 for each point). If we extrapolate this finding to the 127 000 young people currently estimated to have tic disorders we would anticipate a potential cost saving of over £1 million if all young people living with tic disorders in the UK were offered treatment.^9 23^ This saving is based solely on reduced use of other specialist services and does not account for the savings in GP appointments resulting from being ‘bounced back’ from services, school absence or parental time away from work.

### School attendance

The number of days the young people were absent from school, both the total and those considered to be attributed to their tic disorder, is reported in table 3. For each 1-point increase in the YGTSS there is an adjusted mean increase per young person of 0.13 (95% CI 0.08 to 0.18) days off school over 6 months. If only days off school related to tic condition are included, over 6 months there is an adjusted mean increase per young person of 0.08 (95% CI 0.03 to 0.14) days off school for every 1-point increase in the YGTSS.

**Table 3:** Days off school, by total days and those considered related to tics

	Number of participants who had days off (%)	Mean number of days absent for those who report absence (SD)
Total days absent		
Baseline	118/224 (53)	5 (8)
6 months	108/204 (53)	7 (11)
12 months	76/177 (43)	6 (9)
18 months	80/176 (45)	6 (9)
Days off related to tics		
Baseline	45/224 (20)	7 (11)
6 months	37/204 (18)	10 (14)
12 months	24/177 (14)	9 (13)
18 months	18/176 (10)	6 (7)

### Case studies of patient experiences

The case studies below present the experiences of two anonymous patients in trying to reach a diagnosis and treatment for their tic condition; this is presented in online supplemental tables 1 and 2 with the estimated cost associated with each appointment. In summary, from 2017 to 2023, patient A received seven referrals to health psychology, neurology, CAMHS, paediatrics and to a specialist tic clinic, most of which were declined. They visited the GP at least six times and had at least seven CAMHS appointment plus one Choice appointment. Patient A received a diagnosis of tics in 2023 by a CAMHS psychiatrist and was placed on a waiting list for CBIT. Further details and associated costs are found in online supplemental table 1. The total cost of this pathway to the NHS was estimated at £3512.55 and the patient had yet to receive therapy.

Patient B’s account spanned 12 months from March 2022 to 2023. During this time, they were referred to paediatric services (which was rejected), neurology and a specialist tic service (rejected due to being out of area). The family then saw a private neurologist. The patient received multiple conflicting diagnoses, including motor and vocal tics, functional tics, functional neurological disorder and TS. The child no longer attends school and instead attends education at a medical tuition centre. The family are still unclear on the diagnosis and there are no plans to reintegrate the child back into school. The total cost of this pathway to the NHS was estimated at £1594.76 (excluding the private appointments). Further details are found in online supplemental table 2.

Table 4 shows a more appropriate potential pathway for tic conditions whereby patients could be directly referred to a specialist tic clinic for diagnosis by their GP and then access treatment in the form of remotely delivered ERP as provided in the ORBIT trial. This pathway would cost the NHS £1146.76 including treatment for tics, compared with the £3512.55 estimated cost of patient A’s experience, who was unable to access appropriate treatment due to the waiting list for CBIT or £1594.76 for patient B.

**Table 4:** Potential appropriate pathway for tics

Patient requests referral in GP appointment	£42 (PSSRU 2022)
Referred for assessment	£0.76 (PSSRU 2022)
Specialist tic clinic assessment	£949^18^
Access to treatment (eg, 10 sessions of online ERP—ORBIT)	£155
Total	£1146.76

ERP, exposure and response prevention; GP, general practitioner; ORBIT, Online Remote Behavioural Intervention for Tics; PSSRU, Personal Social Services Research Unit.

## Discussion

With the aim of further understanding the impact to healthcare services and education resulting from inadequate tic care pathways and treatment availability, we explored the health service use of children and young people who participated in the ORBIT trial and supplemented this with two case studies. The two case studies demonstrate that the lack of clarity in tic diagnosis is costly to the NHS. The analysis of the ORBIT trial data shows that insufficient treatment is also costly as more severe tics result in additional costs to the NHS and time off school.

The data from the ORBIT trial revealed that patients see several different specialists, resulting in significant costs to the healthcare system. The additional detail provided by our two case studies indicates that the majority of these contacts with specialist services are unlikely to provide adequate support in the assessment or treatment of tic disorders. Data from ORBIT and our case studies also indicate that many children miss school due to their tics. A report from the Department of Education showed that overall absence had a negative impact on attainment, with every additional day missed associated with a lower chance of achieving successful results at General Certificate of Secondary Education (GCSEs).^24^

The findings outline the impact of poor access to appropriate evidence-based treatment and an absence of guidance from national bodies such as NICE on treatment pathways. Our findings highlight a possible variability in the care of individuals with tic disorders which is likely to lead to different outcomes across geographical regions. Our case studies highlight that referrals to specialist tic services may be declined if the referral is out of area. Given the scarcity of specialist tic clinics in England, it is highly likely that most patients will not have access to these services. It is possible that the findings reflect uncertainty in healthcare providers in how best to assess or treat tic cases in the absence of clear NICE guidelines. This is in line with the findings of Marino *et al*^15^ who reported that patients felt their clinician was not knowledgeable in tic disorders. In the absence of evidence-based treatment, it is likely that patients will experience worsening of symptoms, or no symptom improvement,^9^ which in turn is likely to increase the consumption of other NHS systems, resulting in increased waiting lists.

### Clinical implications

A solution to this problem would be to publish NICE guidelines to facilitate a clear assessment and treatment pathway for patients with tic disorders. Providing these guidelines would ensure transparency and equitability in care provision among healthcare professionals and facilitate a standard approach to care. It would also support adequate funding, resources and quality standards being allocated to tic disorders. However, it would be equally important that appropriate evidence-based treatment was available. One solution to this may be found in providing low-intensity, online, remotely delivered therapy, such as that offered in ORBIT. The findings demonstrate here that offering an intervention such as ORBIT has the potential to save a conservative £1 million for every 127 000 young people with tics by reducing burden/use of healthcare services in addition to the clinical benefits to the individual. It is important to note that this possible cost saving is still made even though offering a service such as ORBIT would be adding a new service and offering treatment to children and young people who currently are unlikely to receive any evidence-based therapy for their tics.

### Limitations

Our findings should be interpreted in line with the limitations. These data were taken from the ORBIT trial which was not statistically powered to explore these questions. ORBIT used a parent completed resource use measure, the CA-SUS, to record resource use, which may be subject to bias in memory recall from the parents. The reliability of parent’s reporting of appointments was not validated as part of the ORBIT trial. Furthermore, the costs of the ORBIT platform in the trial may be different from the costs of running ORBIT as service within the NHS. The full economic benefit of establishing ORBIT as a service is being explored in a National Institute for Health Research (NIHR) invention for innovation award (ref: NIHR205467). Our case study data are derived from only two participants, and it is likely that there will be variation in experiences, including cases that may have greater or lesser use of healthcare services as part of their care journey. Where possible, we tried to verify the appointments from the case studies by NHS letters, but this was not always possible, and again the case studies may be subject to bias in memory recall. Furthermore, although participants in ORBIT and the case studies were asked to provide contacts with services because of their tics, it is still likely that participants will need to continue to access other healthcare services as part of ongoing care for comorbidities. Nonetheless, our results provide a critical insight into the impact of inadequate care provision for tic disorders and the variability of patient experience. Further research could explore health resource use using NHS databases to gain a more complete understanding. Our findings are limited to children and young people, although the pathway for adults with tic disorders is likely to also be complex and lengthy, and warrants further investigation.

## Conclusions

Overall, our findings show that young people with tic disorders are likely to engage in substantial use of healthcare resources as a result of inadequate pathways and treatment options. Most young people are unable to access a specialist tic service and case study data indicate that other healthcare services (such as CAMHS or paediatric services) are currently unlikely to have the expertise to support tics. Young people with tics are also likely to miss vital parts of their education. There is a clear need to provide guidelines and services to support people with tics. Our findings indicate that adding a tic treatment service is likely to result in overall cost savings to the NHS as well as providing access to evidence-based treatment, which is currently of limited availability.

## Supplementary material

10.1136/bmjment-2024-301241 online supplemental file 1

## Data Availability

Data are available upon reasonable request.
