# Supplementary material for: Healthcare utilisation and costs associated with poor access to diagnosis and treatment for children and young people with tic disorders
Source: BMJ Ment Health. 2024 Nov 7;27(1):e301241. doi: 10.1136/bmjment-2024-301241 (PMC11552533; doi:10.1136/bmjment-2024-301241)
Supplement: online supplemental file 1 [file bmjment-27-1-s001.pdf]

APPENDIX

Healthcare utilisation and costs associated with poor access to diagnosis and treatment for children and young people with tic disorders

Table 1: Anonymous report from parents of Patient A on their experience (adapted from patient's report)

Date	Description	Cost (In 2023 GBP)
2017	Patient A first saw neurology due to tics appearing. Diagnosis of Pediatric Autoimmune Neuropsychiatric Disorders Associated with Streptococcal Infections (PANDAs). Discharged via letter as PANDAs considered controversial so diagnosis taken away. Tics became more apparent following discharge.	£117 (NHS Reference Costs)
22.3.21	Patient A off school with no voice and sore throat due to vocal tics	
28.5.21	Patient A off school due to exhaustion from vocal tics throughout the night. Dad had to drive Liam around at midnight to calm Liam down.	
12.7.21	Neurology writes to CAMHS requesting Patient A is seen for CBT	£0.76 (PSSRU 2022)
27.8.21	Letter received from CAMHS. Patient A is on waiting list for routine apt.	£0.76 (PSSRU 2022)

Oct 2021	Speak to GP as tics worsening. See if appt for CAMHS can be brought forward	£42 (PSSRU 2022)
14.10.21	Letter received from CAMHS. Patient A doesn't meet criteria to have appointment brought forward.	£0.76 (PSSRU 2022)
15.1.22	Choice assessment via telephone by CAMHS. CAMHS suggested referral to local Health psychology services.	£175 (NHS Reference costs)
12.2.22	Referral to Health psychology service declined. Patient A remains on CAMHS waiting list.	0.76 (PSSRU 2022)
13.6.22	CAMHS video call. Family were told that the service does not work with tics but will address the related anxiety and OCD.	£175 (NHS Reference costs)
19.7.22	CAMHS video call	£153 (NHS Reference costs)
30.9.22	CAMHS appointment face to face sessions start	£383 (NHS reference costs)
Nov 22	3x appointments this month to discuss tics and ask about referrals. GP tries to find a pathway for Patient A but none can be identified. GP completes the funding request but it is declined.	£126 (3x GP appointments, PSSRU 2022)
14.11.22	CAMHS appt again to address anxiety	£383 (NHS reference costs)
15.12.22	2 days off school due to tiredness and neck pain related to tics	

22.12.22	GP asks family to do impact statement about Liam to show how tics impact Liam, he will send this with referrals.	£136 (PSSRU 2022)
5.1.23	CAMHS appt	£383 (NHS reference costs)
16.1.23	CAMHS deliver CBT .	£383 (NHS reference costs)
25.1.23	GP made referrals to neurology, paediatrics and CAMHS again (not sure why as the family are already open to the services)	0.76 (PSSRU 2022)
30.1.23 - 27.2.24	CAMHS deliver behavioural therapy for tics<br><br>Last CAMHS CBT.<br>Treatment seems based around suppressing tics and causing Patient A upset.<br>Therefore after 4 sessions Patient A requests to stop the therapy appointments.	£383 (NHS reference costs) X4 = 1532
31.1.23	Patient A off school due to no sleep related to tics	
Feb 23	declines for referrals to paediatrics and neurology as the family are under CAMHS	0.76 (PSSRU 2022)
16.2.23	Appt with CAMHS psychiatrist. Admits that the family probably know more about Tourette’s than she does. Unsure if she can diagnose and unsure of diagnostic/treatment pathway but says she will find out.	£143 (PSSRU 2022)

2.3.23	Diagnosis of Tourettes made by CAMHS psychiatrist. Prescribes melatonin to help with sleep Currently waiting for CBiT with CAMHS.	£143 (PSSRU 2022)
Total		£3512.55

Note: costs were derived from NHS England. National Schedules of *NHS costs. 2022. Retrieved July 11, 2023, from [https://www.england.nhs.uk/costing-in-the-nhs/national cost-collection/](https://www.england.nhs.uk/costing-in-the-nhs/national-cost-collection/)*

Table 2: Anonymous report from parents of Patient B on their experience (adapted from patient's own report)

Date	**Description**	Cost (In 2023 GBP)
March 2022	First motor tics began. Spoke to psychiatrist at (Children's Eating Disorder Service) who referred Patient B to local Paediatrician Psychiatrist took Patient B out of school and advised not appropriate for Patient B to be there with this new development to her health issues (already had anorexia/self-harm/anxiety)	£113 (PSSRU 2022)
May 2022	Vocal tics began.	
June 2022	Saw Paediatrician at NAMED Hospital who diagnosed motor and vocal tic disorder and referred to neurology	£122 (NHS reference cost)

	department at NAMED hospital. Patient B was placed in a medical tuition centre to try and not fall behind with school.*	
July 2022	A private neurologist diagnosed functional tics and recommended CBIT therapy. Tics attacks began.	£117 (NHS Reference cost) *Cost not included in total as private*
July 2022	CAMHS advised Patient B has been put on wait list for tic therapy with them.	0.76 (PSSRU 2022) *Assumed advised by letter*
September 2022	I contacted the Neurology Department at NAMED hospital to chase referral and was told referral had been rejected due to no service available.	£9 (PSSRU 2022) *Assumed phone call*
September 2022	Parent phoned paediatrician again and asked about neurology referral again due to increase in severity of tics. They contacted department to say that no service is available and neurology at NAMED HOSPITAL or NAMED TRUST would not see Patient B.	£9 (PSSRU 2022) *Assumed phone call*
October 2022	Review with CEDS Psychiatrist who diagnosed Functional Neurological Disorder causing anxiety driven tics	£113 (PSSRU 2022)
December 2022	Follow up with paediatrician (a new one at NAMED HOSPITAL) who gave me a few leaflets	£122 (NHS reference cost)

	and now said Patient B had Tourette's Syndrome rather than functional tics especially since Patient B was now diagnosed with ADHD.	
January 2023	I raised a complaint with ICB and MP due to ongoing issues with being referred, different diagnoses given and no treatment plan or service available. Patient B still out of school with no plan for her education.	
February 2023	In response to complaint, offered an assessment appointment at NAMED hospital with the Tourette Service, which was quickly redacted as we do not live in the area. Appointment was instead given at NAMED hospital with tic therapist.	£129 (PSSRU 2022) *Costed as specialist clinical psychologist assessment appointment likely to be at least 90 minutes*
March 2023	Ongoing – Patient B sees tic therapist regularly. However, this is only due to the complaint raised. This doctor is paid overtime hours in a clinic that isn't for tics at all. This is good for us, but no other child gets these services and it is only because we raised a big complaint. Patient B still does not have a clear diagnosis – it depends which doctor you ask.	£860 (PSSRU 2022) Assume 10 appointments with tic specialist

	Patient B no longer attends school and instead is education at the medical tuition centre still. Patient B will not be reintegrated into school again now and will remain here. There has been a huge detrimental impact to Patient B's education and no doubt Patient B's future prospects.	
Total		£1594.76

Note: **The cost of medical tuition is not considered as this is not due to the lack of pathway for tics but linked to the other health conditions.*

Costs were derived from Jones K, et al. Unit Costs of Health and Social Care (2023). *Unit Costs of Health and Social Care 2022 Manual. Technical report. Personal Social Services Research Unit (University of Kent) & Centre for Health Economics, University of York, 10.22024/UniKent/01.02.100519)*https://kar.kent.ac.uk/100519
